# Size-related variability of oxygen consumption rates in individual human hepatic cells†

**DOI:** 10.1039/d4lc00204k

**Published:** 2024-07-26

**Authors:** Ermes Botte, Yuan Cui, Chiara Magliaro, Maria Tenje, Klaus Koren, Andrea Rinaldo, Roman Stocker, Lars Behrendt, Arti Ahluwalia

**Affiliations:** a Research Centre “E. Piaggio”, University of Pisa Pisa Italy arti.ahluwalia@unipi.it +39 0502217062; b Department of Information Engineering, University of Pisa Pisa Italy; c Department of Organismal Biology, Science for Life Laboratory, Uppsala University Uppsala Sweden lars.behrendt@scilifelab.uu.se +46 184712591; d Department of Materials Science and Engineering, Science for Life Laboratory, Uppsala University Uppsala Sweden; e Aarhus University Centre for Water Technology, Department of Biology, Aahrus University 8000 Aarhus Denmark; f Laboratory of Ecohydrology ECHO/IIE/ENAC, École Polytechnique Fédérale de Lausanne Lausanne Switzerland; g Department of Civil, Environmental and Architectural Engineering, University of Padova, Padova Italy; h Institute for Environmental Engineering, Department of Civil, Environmental and Geomatic Engineering, ETH Zurich Zurich Switzerland

## Abstract

Accurate descriptions of the variability in single-cell oxygen consumption and its size-dependency are key to establishing more robust tissue models. By combining microfabricated devices with multiparameter identification algorithms, we demonstrate that single human hepatocytes exhibit an oxygen level-dependent consumption rate and that their maximal oxygen consumption rate is significantly lower than that of typical hepatic cell cultures. Moreover, we found that clusters of two or more cells competing for a limited oxygen supply reduced their maximal consumption rate, highlighting their ability to adapt to local resource availability and the presence of nearby cells. We used our approach to characterize the covariance of size and oxygen consumption rate within a cell population, showing that size matters, since oxygen metabolism covaries lognormally with cell size. Our study paves the way for linking the metabolic activity of single human hepatocytes to their tissue- or organ-level metabolism and describing its size-related variability through scaling laws.

## Abstract 2

Human hepatocytes confined in microwells cooperate in oxygen sharing and their size-metabolism covariance is lognormal.

## Graphical abstract

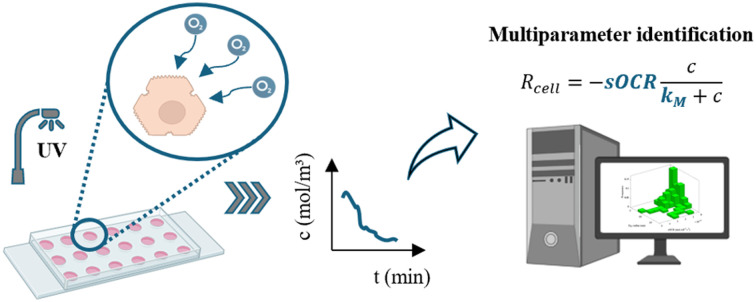

## Introduction

Biological variability (*i.e.*, the fluctuation of physiological traits among individuals of the same population, also referred to as biological noise) is ubiquitous and can impact phenomena such as metabolic scaling and resilience to environmental perturbations.^1,2^ Often, variability is not confined to one parameter but instead is characterized by an interplay between multiple variables within an organism or ecosystem. For instance, it has been suggested that the covariance between the size and metabolism of individuals influences the ability of organisms to react to external stimuli (*e.g.*, toxins or drugs) and may explain patterns in homeostatic control.^1,3,4^ Joint variations between physiological parameters can also impact the susceptibility of organisms to diseases and affect their overall health.^5^ Biological variability has been extensively investigated at the molecular level (transcription and expression), but less so at cellular and organismal levels. Investigating single cells, instead of tissues or organs, offers the opportunity to characterize variability between individuals to infer dynamics occurring at higher scales of complexity. Single cells also provide a suitable testbed to determine intrinsic size-related variability and its role in metabolic scaling, which has been highlighted as a criterion for translating biological parameters from micro-scale *in vitro* systems to *in vivo* contexts.^1,6,7^

As oxygen (O_2_) is at the heart of aerobic metabolism,^8,9^ several studies have measured O_2_ consumption in individual mammalian cells.^10–13^ However, the accuracy, reproducibility, and throughput of O_2_ measurements at this scale remain challenging. Mammalian cells possess the ability to modulate their O_2_ consumption according to its availability, a process that, in turn, is influenced by numerous factors (*e.g.*, height of culture medium and cell density).^14,15^ However, many studies assume that cells possess a constant (zero-order) consumption rate which depends only on cell phenotype. The O_2_ consumption rate (*R*_cell_, in mol s^−1^) of a single cell as a function of the surrounding O_2_ concentration (*c*, in mol m^−3^) is typically represented by the Michaelis–Menten (MM) model^16–18^*via* two parameters: the maximal consumption rate (sOCR, in mol s^−1^) and the MM constant (*k*_M_, in mol m^−3^).1
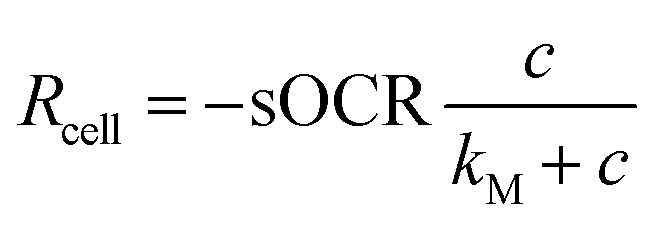
*k*_M_ corresponds to the concentration at which the consumption rate is half of its maximum value. At high O_2_ levels (*c* ≫ *k*_M_), the cellular consumption rate saturates at its maximum value (*i.e.*, *R*_cell_ ≅ −sOCR). On the other hand, if *c* ≪ *k*_M_, the cellular uptake rate depends on the O_2_ concentration as 
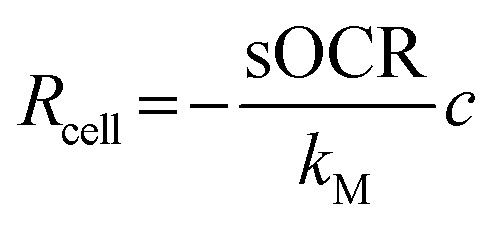
. Hence, a cell with a low *k*_M_ value consumes O_2_ maximally even at low concentrations, whereas a cell with a high *k*_M_ has a low O_2_ uptake efficiency (*i.e.*, O_2_ levels must be high to achieve near-maximal consumption rates).

Despite the widespread application of eqn (1) in predicting how cells adapt to ambient O_2_, MM parameters for mammalian cells have rarely been reported. Some studies have investigated cell cultures either as 2-dimensional (2D) monolayers or 3-dimensional (3D) constructs (herein referred to as *aggregates*, independent of dimensionality), but to date MM parameters for individual mammalian cells have not been measured. Here we present a systematic approach to conduct single-cell measurements of O_2_ consumption as described by the MM model. Our primary objective was to estimate the MM parameters (*i.e.*, sOCR and *k*_M_) and explore their size-related variability in a human hepatic cell line, HepG2.^19^ Using custom glass microwell devices coated with luminescent O_2_-sensitive optode materials,^20,21^ we isolated single cells or *clusters* of a few cells (from two to seven units confined in the same microwell but not necessarily adjacent, unlike in 2D or 3D aggregates) under precisely controlled experimental conditions. Automated fluorescence microscopy was used to perform time-series imaging of the wells to extract cell sizes and O_2_ concentration profiles from individual wells. A multiparameter identification procedure^22^ was applied to determine MM parameters from these profiles. Through this approach, we were able to estimate single-cell size and MM parameters for O_2_ consumption as a joint probability distribution and so describe their correlated variability.

This quantitative description of single-cell O_2_ consumption allows probing the biophysical basis of cooperative metabolic dynamics, which are only detectable at higher levels of organization. Furthermore, the identification and characterisation of the covariation of size and O_2_ consumption parameters for single cells can help in understanding how these dynamics are linked to behaviours that emerge in tissues and organs. It also provides a means for investigating the origins of allometric scaling between size and metabolic rates of whole organisms, a subject of long and wide-ranging debate.^3^

## Materials and methods

### Microwell devices for single-cell isolation and oxygen sensing

**Fig. 1:**
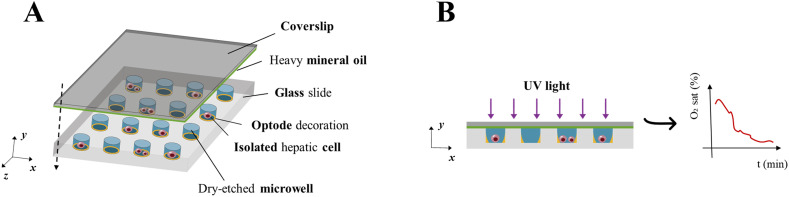
Schematic of the assembled glass microfluidic device. (A) Microwells coated with optode chemistry are seeded with hepatic cells suspended in culture medium and can contain a single cell or a few cells. Subsequently, the device is overlaid with a glass coverslip coated in heavy mineral oil, which reduces lateral (*i.e.*, inter-well) O_2_ diffusion so that the microwells effectively reach hypoxic conditions. (B) Once sealed, the system is exposed to short pulses of UV light and the resulting luminescence emission from optodes is detected over time to derive O_2_ concentrations across microwells. Inset: a typical O_2_ concentration profile obtainable by monitoring optode response in an individual microwell where at least one cell is settled.

The microwell array consists of a standard borosilicate glass slide with geometrically arranged microwells (100 columns × 250 rows, *n* = 25 000 microwells) fabricated *via* standard UV lithography and dry etching techniques. It was custom-built to allow the isolation of single cells or small clusters of cells in each microwell (see the schematic in Fig. 1 and S2†). The microfabrication procedure for this device is summarised in the ESI† (Fig. S1), and Table S1† reports relevant technical features of the dry etching process and geometric specifications of the array customized to match the size of human hepatic cells and to minimize optical and diffusional crosstalk between microwells.

### Optode sensor composition, deposition and calibration

#### Composition

Quenching-based luminescent optode materials were chosen for O_2_ sensing as they are characterized by a high spatial resolution and short response time, as required for single-cell O_2_ consumption rate measurements.^23,24^ The deposited optode material was composed of platinum(ii)-5,10,15,20-tetrakis-(2,3,4,5,6-pentafluorophenyl)-porphyrin (PtTFPP), polystyrene (PS) and MACROLEX® yellow 10GN (MY) dissolved in toluene. Here, PtTFPP is the O_2_-sensitive dye whereas MY acts as a reference (*i.e.*, O_2_-insensitive) dye. Both dyes are excited using UV light (*λ* = 396 nm) and emit red luminescence (PtTFPP, *λ* ≥ 650 nm) or green luminescence (MY, *λ* = 507 nm) in an O_2_ concentration-dependent manner^24^ or at a constant intensity, respectively. Since fluctuations of the emission intensity due to environmental factors independent of the O_2_ concentration affect both dyes, while only PtTFPP is sensitive to O_2_ level variations, the ratio of red-to-green emission intensities (*R*) represents a robust O_2_-dependent signal, with minimal noise from environmental artefacts (*e.g.*, sensor bleaching).^25,26^ With appropriate calibration, *R* thus allows for relating the signal to O_2_ content within individual microwells.

#### Deposition

**Fig. 2:**
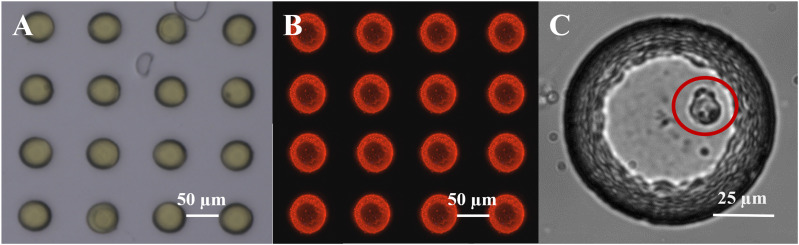
O_2_ sensing in glass microwells. (A) Brightfield image of individual microwells with deposited optode chemistry (yellow) in the custom-built glass microwell array. (B) Corresponding luminescent response (red) upon exposure to excitation light. (C) Image of a single hepatic cell in a 100 μm diameter microwell.

To deposit the optode material, a 10% w/v solution of PS in toluene containing 0.15 g L^−1^ of both MY and PtTFPP was spread on the glass microwell array *via* a thin film applicator. The homogeneity of the optode coating was assessed by profilometry (see ESI1† for details), which revealed that it had a nominal thickness of 5 μm and was most uniform in the central region of the array. Therefore, we prioritized this central area (containing at least 200 microwells) for seeding cells and investigating their O_2_ consumption. As the deposited optode material is hydrophobic, the array was briefly treated with O_2_ plasma (Zepto, Diener electronic, Ebhausen, Germany) for 10 s at 0.4 mbar and 50% intensity to promote filling of microwells with culture medium and assist in cell adhesion. This plasma treatment did not affect the response of optode materials to O_2_. To avoid undesired background fluorescence, the optode material deposited outside of the microwells was removed using a scalpel. This process resulted in two regions with different wettability, *i.e.*, (i) hydrophilic microwells containing a layer of optode material (yellow regions in Fig. 2A) surrounded by (ii) hydrophobic glass (gray regions in Fig. 2A).

#### Calibration

Following optode deposition, a calibration curve was constructed by averaging data acquired from 216 central microwells. To perform calibration measurements, the optode-coated array was placed into a gas-impermeable chamber with a transparent window for image acquisition. The chamber was placed in a microscopy incubator (Okolab srl, Pozzuoli, Italy) maintained at 37 °C, to avoid temperature fluctuations which might influence optode responses (Fig. S2†). Optode emissions were recorded *via* a fully automated fluorescence microscope (Nikon Ti2-E, Nikon, Tokyo, Japan) equipped with an RGB camera (DFK 33UX264 colour industrial camera, The Imaging Source) and a LED excitation light (Spectra X, Lumencor, OR, USA).

Calibration was performed introducing gases at five different levels of O_2_ saturation by combining compressed air and nitrogen (N_2_) through a gas mixer set up (Red-y smart series mass flow meters, Voegtlin GmbH, Muttenz, Switzerland). This system enabled modulation of the partial pressures of the mixture components by tuning the flow rate from their sources. The stability of air saturation within the sealed chamber was verified by monitoring it with a calibrated optical microsensor (OXR250, Pyroscience GmbH, Aachen, Germany). Images of single microwells were acquired with a 40× objective. All other imaging parameters were set as listed in Table 1 for monitoring cellular O_2_ consumption. A simplified form of the two-site model (eqn (2))^27–29^ was then fitted to collected datapoints in order to derive average calibration parameters for the specific array – *i.e.*, the Stern–Volmer (SV) constant^24^ of the primary site for O_2_ quenching on the dye (*k*_SV_, expressed in 1/% air sat.) and the fractional contribution (*f*) of such primary site to the red-to-green ratio in totally anoxic conditions (*R*_0_, measured by exposing the device to pure N_2_).2
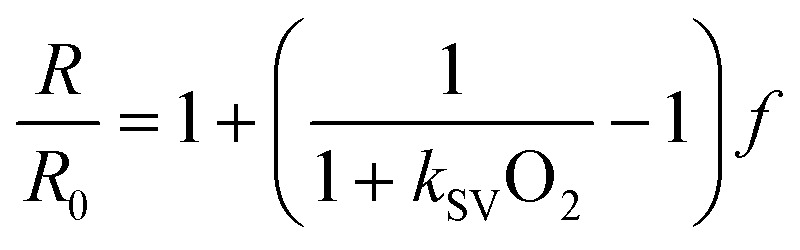
In eqn (2), O_2_ (% air sat.) denotes the O_2_ saturation level in the gas mixture. Refer to the ESI† (section ESI2) for further details on the calibration model.

**Table 1:** Experimental parameters for O_2_ sensing. Parameters refer to the Nikon Ti2-E automated fluorescence microscopy system used for O_2_ consumption measurements

Parameter	Numerical value
Magnification	10×
Large image size (*n* × *m*)	6 × 6
Time scale (*t*_end_)	1 h
Time resolution (Δ*t*)	20 s
Excitation light wavelength	396 nm
Excitation light intensity	20% of maximum intensity^a^
Excitation duration	50 ms

a To achieve optimal saturation of the sensing dye, the intensity of the excitation light was coherently adjusted for each round of calibration and subsequent experiment.

Calibration curves were also estimated in liquid phase (*i.e.*, cell culture medium) and compared to those obtained for corresponding microwell arrays using gases (data not shown). No significant differences emerged between liquid phase and gas phase calibrations, and hence we routinely relied on the more convenient gas calibration prior to each experiment. Further, we ensured that optode bleaching was negligible in the timeframe of our single-cell O_2_ consumption tests (see ESI3† for details). Finally, experiments were conducted to determine the extent of optical crosstalk between microwells and to define inter-well distance and cell occupancy to minimize it (see ESI4† for details).

### Single-cell oxygen measurements

#### Cell preparation

Human hepatic cells from hepatocellular carcinoma (HepG2 cells, ATCC, Manassas, Virginia, USA) were maintained in T25 flasks (Sarstedt, Numbrecht, Germany) under standard conditions (37 °C, 95% humidity, 5% CO_2_). Fresh Dulbecco's Modified Eagle Medium (DMEM – Sigma-Aldrich, St Louis, Missouri, USA) supplemented with 10% v/v foetal bovine serum (FBS – Sigma-Aldrich) was supplied every 3 days. Before experiments, cells were detached with trypsin–EDTA (Lonza, Basel, Switzerland).

After optode calibration, the central area of the microwell array was overlaid with a thick poly-dimethyl-siloxane (PDMS) frame into which 500 μL of cell suspension in DMEM was pipetted. This allowed control of the seeding density and ensured that cells were confined to the region where the optode coating was determined to be homogeneous. Experiments were performed with different seeding densities, ranging from 2 × 10^3^ cells per mL up to 10^6^ cells per mL. The former resulted in an optimal trade-off between an acceptable number of microwells containing single cells and minimal lateral O_2_ diffusion between neighbouring microwells. Following cell seeding, the device was incubated overnight to allow cell adhesion to the microwells (Fig. 2C).

#### Monitoring single-cell consumption

Immediatly before O_2_ consumption experiments, the culture medium within the PDMS frame was changed and 60 μL of fresh DMEM was dispensed into the microwells. Then, the PDMS frame was removed and microwells seeded with cells were covered with a coverslip, which was affixed to the array *via* both paperclips and magnets (Fig. S1†). A thin coating of heavy mineral oil (Sigma-Aldrich) was applied to the underside of the coverslip to isolate the microwells from environmental O_2_ (Fig. 1) and to define the initial and boundary conditions of the system. Mineral oil has a significantly lower O_2_ diffusion coefficient than aqueous media^30^ and effectively seals off the array from ambient O_2_, allowing a hypoxic steady state to be reached – a necessary condition to properly characterize the MM consumption kinetics, particularly *k*_M_ (see subsection *Modelling single-cell consumption).* The ratio of mineral oil to cell culture media was optimized to ensure maximal phase separation and minimize lateral (*i.e.*, inter-well) O_2_ diffusion and, at the same time, guarantee that cells are exposed to the medium phase within microwells (see ESI5† for details). Image acquisition was started immediately after device assembly to ensure rapid monitoring of O_2_ dynamics (*i.e.*, from the initial condition of maximum O_2_ in the medium to the achievement of stationary hypoxia, defined as *c* ≤ 0.04 mol m^−3^ (ref. 31)). Measurement duration and sampling frequency were set according to instrument limits and modelling considerations (see subsection *Modelling single-cell consumption*). The O_2_ dynamics associated with cell consumption were measured by repeatedly scanning the central area containing cells, using large area scanning mode. Briefly, this mode allows for the acquisition of a matrix of images (referred to as *large image*) covering 216 microwells, that are serially acquired according to a raster scanning path defined by means of the spatial coordinates of its centroid. The large image acquisition parameters are listed in Table 1.

Following each experiment, 30 μL of trypan blue dye (0.4% w/v solution, Sigma-Aldrich) was gently pipetted through the gap between the microwell array and the coverslip. Trypan blue was left to diffuse over the array for about 10 min, then a brightfield large image was acquired setting the same scanning pathway as used during experiments. This procedure enabled identifying *N*_cell_ in each microwell and estimating cell size (*i.e.*, the projected area) using ImageJ.^32^

#### Determination of concentration profiles

Time series of large images were processed exploiting algorithms purposely developed in Matlab (R2021b, MathWorks, Massachusetts, USA). Briefly, each RGB image was segmented by means of customized thresholding to distinguish PtTFPP-decorated microwells from the background. This allowed for the computation of the pixel-by-pixel *R*. Finally, for each microwell, *R* profiles were determined over time by averaging over pixels belonging to the same microwell. This allowed for conversion of *R* signals into O_2_ concentrations using previously established calibration curves.

#### Modelling single-cell consumption

Leveraging analytical considerations and bearing in mind that neither cell location nor the number of cells per well (*N*_cell_) can be determined *a priori*, we established the duration (*t*_end_) and sampling frequency (*f*) of measurements based on the experimental setup. From a modelling point of view, each microwell is a region of the space governed by the reaction–diffusion equation (eqn (3)):3
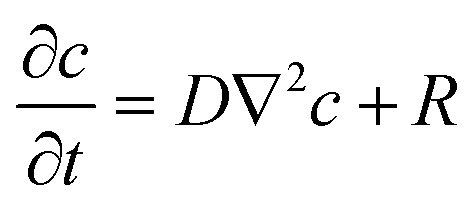
where *c* = *c*(*x*, *y*, *z*, *t*) (mol m^−3^) is the O_2_ concentration field in the microwell, *D* (m^2^ s^−1^) is the diffusion constant of O_2_ and *R* = *R*(*x*, *y*, *z*, *t*) (mol m^−3^ s^−1^) is the O_2_ production/consumption rate per unit volume. In the case of consumption, *R* is negative and can be described as a function of the single-cell MM consumption rate (*R*_cell_) defined in eqn (1). Thus:

4 *R* = *ρ*_cell_*R*_cell_

where *ρ*_cell_ (cells per m^3^) is the cell density in the microwell volume.

Given that cell and microwell sizes are comparable, and assuming that O_2_ consumption is uniform in the cell volume – and hence in the well domain – the cell density can be expressed as *ρ*_cell_ = *N*_cell_/*V*_well_ (with *V*_well_ indicating the microwell volume, Table S1†).

As the heavy mineral oil layer is considered impermeable to O_2_, there is no flux at the oil/air boundary, likewise at the microwell walls or – given the homogeneity of consumption in the volume – within the domain. These assumptions imply that the O_2_ concentration field in the microwell depends only on time (*c*(*x*, *y*, *z*, *t*) = *c*(*t*)), and the governing equation can be simplified as 
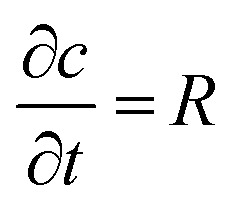
. In these conditions, a suitable duration for monitoring O_2_ consumption to hypoxia within each microwell was estimated based on the characteristic reaction time, *τ*_r_:5
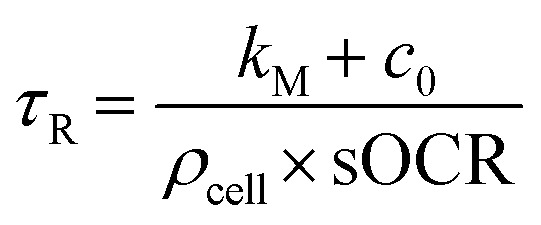
where conditions of O_2_ saturation (*i.e.*, *c* = *c*_0_, see Table 2) and *N*_cell_ = 1 were assumed. These are cautious choices leading to the longest *τ*_r_ possible for the system, guaranteeing that the consumption dynamics are fully captured. Typical literature values were then used for sOCR and *k*_M_,^6,18,33,34^ giving *τ*_r_ = 678.4 s. Thus, experiments were set with *t*_end_ ≥ 5*τ*_R_ and *f* ≥ 10/*τ*_R_.

**Table 2:** Initial condition and range of parametric sweep implemented for simulating O_2_ dynamics within a microwell where *N*_cell_ cells have settled

Parameter	Numerical value	Description
*c*_0_	0.2 mol m^−3^ (ref. 18)	Initial condition of O_2_ saturated-culture medium
sOCR	[10^−18^; 10^−16^] mol s^−1^ per cell (deduced from ref. 18 and 35)	Initial range of parametric sweep
*k*_M_	[10^−3^; 10^−1^] mol m^−3^ (deduced from ref. 17, 18 and 35)	Initial range of parametric sweep

**Table 3:** Values of sOCR and *k*_M_ from previous studies as bulk averages of hepatic cell aggregates. Data are expressed as mean ± standard deviation. For consistency with the literature, values from this and our previous works are reported using the same statistics. For studies investigating different cell densities,^15,22^ values corresponding to the median cell density are reported. For references which consider zero-order consumption kinetics,^33,34,36,37^*k*_M_ values are not applicable (NA)

Aggregate type	sOCR (×10^−18^ mol s^−1^ per cell)	*k*_M_ (×10^−3^ mol m^−3^)	Reference
Isolated single cell	13.35 ± 10.05	76.40 ± 52.60	This study
Cell-laden hydrogel (3D)	69.00 ± 0.15	6.20 ± 0.06	15
Cell monolayer (2D)	43.85 ± 27.41	23.50 ± 2.20	22
Cell-laden spheroid (3D)	78.07 ± 11.45	3.78 ± 1.52	22
Cell-loaded hollow fibers (bioartificial liver)	13.00 ± 5.89	NA	34
Cell monolayer (2D)	55.00 ± 9.85	NA	33
Cell-laden hydrogel (3D)	24.00 ± 7.13	NA	36
Microcarrier (3D)	75.00 ± 18.68	NA	37

### Kinetic parameter identification

Experimentally measured O_2_ concentration profiles constituted the input datasets for the multiparameter identification algorithm reported in.^22^ Briefly, values of sOCR and *k*_M_ were estimated comparing the O_2_ dynamics measured in each microwell containing cells to those predicted *in silico* by modelling the system according to eqn (3) and (4). A model governed by the dimensionless form of eqn (3) was iteratively solved for each specific microwell, taking *N*_cell_ from the trypan blue-stained image and parameters listed in Table 2 into account.6
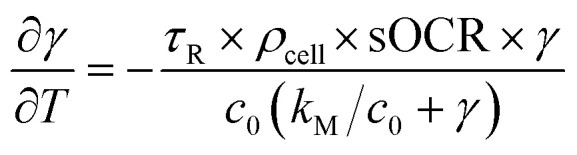


In eqn (6), *γ* = *c*/*c*_0_ and *T* = *t*/*τ*_R_ are the non-dimensional concentration and time, respectively. Considering eqn (5), the dimensionless equation implemented for simulating O_2_ consumption in the well is the following:7
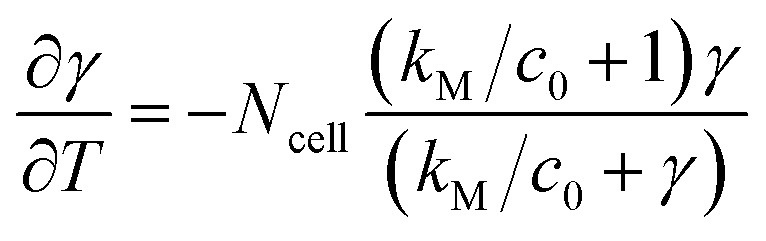
The multiparameter identification algorithm described in ref. 22 was used to estimate the MM parameters through eqn (7). Although the equation does not explicitly depend on sOCR, the latter determines the time scale of the solution, given the definition provided for the dimensionless time *T*.

### Statistical analysis and software

Numerical values of consumption parameters identified for microwells as a function of *N*_cell_ were compared by means of a non-parametric Kruskal–Wallis test and pairwise *post hoc* Dunn's multiple comparisons. Non-linear correlation (*i.e.*, non-parametric Spearman coefficient) of sOCR and *k*_M_ with respect to *N*_cell_ as well as with each other was also tested. Overall statistical differences between the MM parameters estimated here and typical literature values were assessed for both sOCR and *k*_M_ using a non-parametric Wilcoxon signed-rank test. Furthermore, the joint distribution of single-cell size and sOCR was tested for both normality and lognormality *via* the Henze–Zirkler multivariate normality test performed on the original and log-transformed dataset, respectively.

Image processing, simulation of O_2_ transport and consumption and multiparameter identification were implemented in Matlab (R2021b), while GraphPad Prism (version 7, GraphPad Software, California USA) was used to perform all statistical analyses.

## Results

**Fig. 3:**
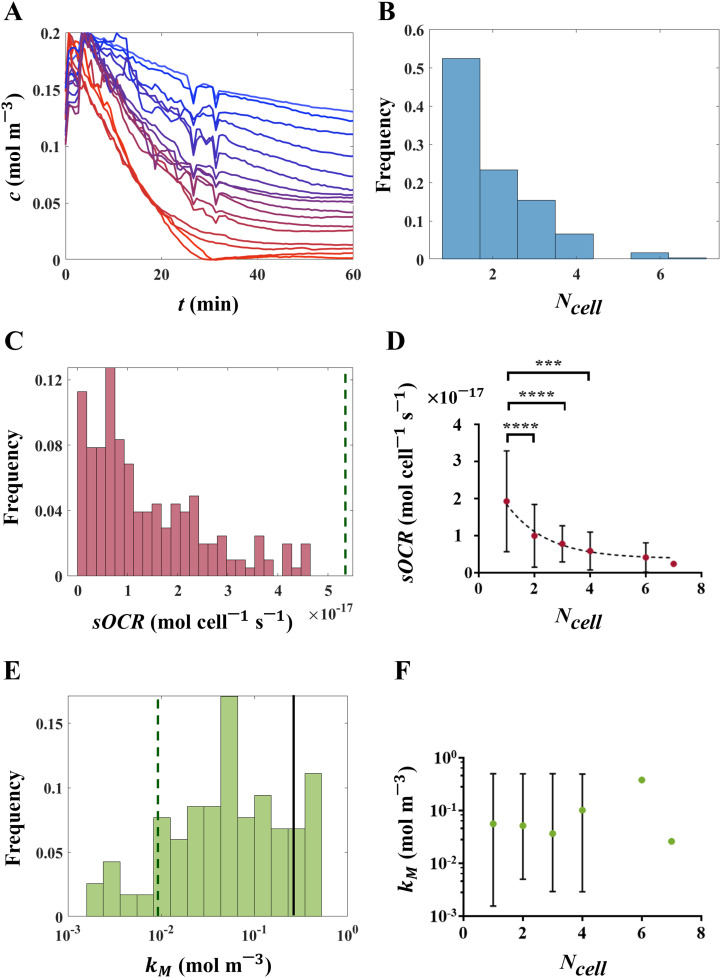
MM kinetic parameters in isolated hepatic cells determined through a multiparameter identification procedure. (A) O_2_ concentration profiles measured in a microwell array. (B) Occupation (*N*_cell_) frequency of cells in microwells. (C) Relative frequency of sOCR in occupied microwells. (D) Correlation between the number of cells per microwell and the corresponding sOCR value (*r* = −1 with *p* = 0.0028), over all occupied microwells. The black dashed curve is a weighted fit of results (one phase exponential decay, *R*^2^ = 0.9722). Pairwise statistical differences computed using Dunn's *post hoc* multiple comparisons are also reported (* *p* < 0.05, ** *p* < 0.005). (E) Relative frequency of *k*_M_ in occupied microwells (expressed in logarithmic scale). (F) No correlation was observed between *k*_M_ and the number of cells per well (*r* = −0.8286 with *p* = 0.0583). The vertical dark green dashed lines in (C) and (E) denote median literature values of sOCR and *k*_M_,^15,18,22,33,34^ respectively (see Table 3 for a complete list of reported values), while the solid black line in (E) indicates the O_2_ saturation level in water (*c*_0_). In (D) and (F), data are reported as median ± range.

Using glass microwell devices (Fig. 2A) sealed *via* heavy mineral oil in conjunction with O_2_ sensitive optode chemistry (Fig. 2B) we measured a total of 1080 microwells across five independent experiments. Of these, 227 microwells (∼21%) contained at least one hepatic cell whose O_2_ consumption dynamics (Fig. 3A) could be recorded without interference from neighbouring wells (see ESI,† section ESI4). The distribution of the number of cells per well (or occupation, *N*_cell_) is reported in Fig. 3B. Notably, in more than half of the cases in which cells were present in a microwell (*i.e.*, 119 measurements, ∼11% of all investigated microwells), a single cell was probed (Fig. 2C), while a maximum of seven cells per well was recognized only once.

### Oxygen consumption kinetics and number of cells per well

O_2_ profiles determined from individual or multiple cells were used to compute sOCR and *k*_M_ through a multiparameter identification algorithm.^22^ Both MM parameters are reported as overall probability distributions (Fig. 3C and E) and as a function of *N*_cell_ (Fig. 3D and F). A Kruskal–Wallis test highlighted significant differences among sOCR values determined for microwells containing different numbers of cells (*p* < 0.0001). Specifically, we found that sOCR decreases with increasing *N*_cell_ (Fig. 3D, Spearman coefficient *r* = −1 with *p* = 0.0028); this suggests that cells adjust their sOCR in the presence of neighbouring cells sharing the same microenvironment. Notably, although our sOCR values are similar to previous estimates for hepatocytes cultured in a hollow fibre bioartificial liver,^34^ they are significantly lower (median sOCR = 1.1 × 10^−17^ mol s^−1^ per cell) than most of those reported in the literature for hepatocyte monolayers or 3D aggregates (median sOCR = 5.5 × 10^−17^ mol s^−1^ per cell, Wilcoxon test, *p* < 0.0001, Fig. 3C).^6,15,18,22,33,36,37^

Our measurements of *k*_M_ show a wide distribution, covering three orders of magnitude (Fig. 3E), with a significantly higher median (5.1 × 10^−2^ mol m^−3^) compared to values previously reported for 2D and 3D hepatic constructs (6.2 × 10^−3^ mol m^−3^ – Wilcoxon test, *p* < 0.0001).^15,22^ Approximately 34% of the measured *k*_M_ values are comparable to or even higher than the O_2_ saturation level in water (*c*_0_ = 0.21 mol m^−3^), suggesting that, once isolated, about a third of the cells do not approach their maximal consumption rate but instead follow first order kinetics. Due to the large variability of *k*_M_, neither a significant correlation with *N*_cell_ (Spearman coefficient *r* = −0.8286 with *p* = 0.0583) nor statistical differences among its medians for different *N*_cell_ values (Kruskal–Wallis test, *p* = 0.2075) were detected (Fig. 3F).

**Fig. 4:**
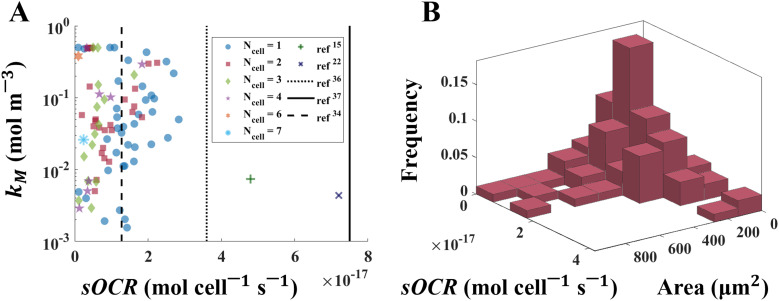
Mutual dependency of MM parameters and the size-related variability of O_2_ consumption in single human hepatic cells. (A) Scatter plot of measured sOCR and *k*_M_ values. The Spearman coefficient indicates that the two parameters are weakly correlated (*r* = 0.5864). Each data point is denoted by a marker having shape and colour which depend on the corresponding value of *N*_cell_ for that data point. The green plus^15^ and the dark blue cross^22^ are median bulk values from the literature (see Table 3). Black vertical lines indicate sOCR values from previous studies assuming zero-order kinetics for O_2_ consumption (dotted: ref. 36, solid: ref. 37, dashed: ref. 34). Note that *k*_M_ values are reported in logarithmic scale. (B) Joint distribution of single-cell size (*i.e.*, projected area) and O_2_ metabolism (*i.e.*, sOCR), expressed as relative frequency of occurrence.

To help distinguish mutual dependencies of sOCR and *k*_M_, MM parameters for all microwells investigated are presented in a scatter plot (Fig. 4A). Data points referring to microwells populated by the same number of cells are clustered along the sOCR axis, while no noticeable separation among groups of points corresponding to different values of *N*_cell_ with respect to *k*_M_ is observed. A correlation analysis suggests that the two MM parameters depend on each other (Spearman coefficient *r* = 0.5864 with *p* < 0.0001), albeit less significantly than in previous observations for 2D and 3D aggregates of hepatic cells (*e.g.*, *r* = 0.7857, ref. 22). For comparison, bulk values for the two kinetic parameters, averaged over several cells as reported in the literature,^6,15,18,22,33,34,36,37^ are also indicated in Fig. 4A and listed in Table 3.

### Joint measurements of single-cell size and metabolism

Immediately after each experiment, HepG2 cells were stained with Trypan Blue, which allowed determining single-cell sizes from projected cell areas. From this data, we estimated the joint distribution of single-cell size and sOCR (Fig. 4B). The Henze–Zirkler test (*α* = 0.05) demonstrated that the sample can be described by a lognormal joint probability density function (*p* = 0.0633). Further confirmation of lognormality was provided by independent optical measurements of dry mass of individual HepG2 cells – performed by means of quantitative phase imaging – which also displayed a marginal distribution with a lognormal shape (see ESI6†).

## Discussion

Here we report on the characterization of O_2_ metabolism in single human hepatic cells through an integrated *in vitro*–*in silico* approach. One or a few cells were seeded in microfabricated microwells coated with O_2_-sensitive optodes and sealed from ambient air, enabling the precise measurement of O_2_ consumption over time. These data were then exploited in a multiparameter identification algorithm^22^ to characterize the cellular O_2_ consumption kinetics according to the MM model (eqn (1)). As shown in Fig. 3C and Table 3, we demonstrate that isolated HepG2 cells have lower sOCR values compared to previously reported ones for 2D or 3D aggregates of the same cell type in comparable environmental conditions (except for ref. 34), including those obtained in our previous studies.^15,22^ Interestingly, the sensing principle (*i.e.*, fluorescence quenching) exploited in ref. 15 and 22 is the same as that of the optodes used here, while the other data in Table 3 refer to O_2_ concentration measurements performed using commercial electrochemical sensors (*e.g.*, Clark-type electrodes). This suggests that cells change their O_2_ consumption when isolated as individuals or in clusters of a few cells, reducing their maximal consumption rate (*i.e.*, sOCR). Furthermore, the decrease in sOCR with increasing microwell occupancy (*N*_cell_) shows that changes in metabolism occur as a function of both the local O_2_ concentration – as per the MM equation – and the presence of neighbouring cells (Fig. 3D) – manifested as a reduction in sOCR. This behaviour mirrors observations on hepatocytes in 2D and 3D aggregates and could be interpreted as an effect of cooperation between individual cells which are not aggregated but coexist in a microenvironment where a limited resource is shared. However, contrary to our previous observations for hepatic cells in 2D and 3D, the adaptive behaviour – that is the modulation as a function of the number of neighboring cells, if any – in isolated cells appears not to affect the O_2_ uptake efficiency, since *k*_M_ is not dependent on *N*_cell_ (Fig. 3F).

In fact, the correlation between the two parameters is weak (Fig. 4A) and less significant than in 2D or 3D aggregates.^22^

It is worth noting that the identification of *k*_M_ is influenced by the ability of the microwell system to effectively achieve hypoxic steady-state conditions. Thus, although the optodes are highly sensitive at low O_2_ concentrations (see ESI2†), even slight variations of stationary O_2_ levels can influence the estimation of *k*_M_ and widen its dispersion. Indeed, precisely characterizing *k*_M_ is a well-known challenge due to the sensitivity of estimated values to experimental conditions,^22,38,39^ which might ultimately mask potential trends. Nonetheless, the estimates of sOCR reported here are statistically robust and not impacted by the uncertainty in *k*_M_, as shown in Fig. 3D.

In ref. 22 we showed that the MM parameters can be combined to define an uptake coefficient – a measure of the surrounding area per unit time in which a cell is able to perceive and consume O_2_ – which can be related to a proximity index – PI, expressing the extent of cell packing within the aggregate.^22^ The uptake coefficient increases to a saturating threshold along with PI in cell aggregates. In the microwells, in the limit PI → 0 for a single cell, while it is of the order of magnitude of 10^−3^ μm^−1^ for cell clusters (see SM7 for the analytical details), leading to uptake coefficients coherent with the values of sOCR and *k*_M_ we report here (Fig. 3, Table 3). This suggests that the lower values of sOCR with respect to cell aggregates are a consequence of limited packing and inter-individual interaction (*i.e.*, aggregation) in microwells.

Together with our previous findings,^22^ this study lays the foundations toward a refinement of the current formulation of the MM model, involving the mutual dependency of sOCR and *k*_M_ through the extent of cell packing (*i.e.*, PI). This might be beneficial for predicting O_2_ metabolism in *in vitro* liver systems and consequently promote the design of tissue models with enhanced resemblance to their *in vivo* counterpart. Furthermore, these results may help in the mechanistic explanation of liver zonation^40^ and provide useful insights for the development of novel and more effective therapeutic treatments for hepatic diseases.

The throughput of our approach also allowed us to measure the correlated variability of cell size and maximal O_2_ consumption rate (*i.e.*, sOCR) and describe their joint frequency distribution (Fig. 4B). This outcome successfully overcomes the challenge of jointly investigating individual size and metabolism, a hurdle previously encountered by our group and others.^3^ Although further experiments are required to fully capture the covariance between single-cell size and O_2_ consumption,^1^ our study provides the first size-metabolic rate distribution based on joint experimental datasets reported so far. Notably, our analysis demonstrates that the sample is extracted from a population characterized by a lognormal multivariate function. Lognormally-distributed marginal probabilities have been commonly observed for organismal sizes,^41^ but they have not been explicitly reported for metabolic rates. Their covariation demonstrates that size is a determinant of O_2_ consumption in single cells. The lognormality suggests that most cells are relatively small and typically associated with low values of sOCR, while larger cells are less frequent and unlikely to consume O_2_ rapidly. This result is also supported by previous experimental investigations on eukaryotic cells,^42–44^ highlighting that there exists an optimal range of sizes which allows for optimized single-cell metabolic activity. This implies that – although the mitochondrial count per cell has been reported to increase linearly with size^44^ – the largest individuals within a cell population are less efficient from a functional point of view and hence rarely observable.

Characterizing the covariance of single-cell size and metabolic parameters – as achieved here – is crucial to comprehensively describe the O_2_ consumption dynamics of human hepatocytes in isolation. It also paves the way to link these dynamics to the tissue or organ levels and to interpret behaviours emerging at higher scales such as cooperation or size-related (allometric) scaling. A direct application of this work is the development of engineered cellular systems capable of recapitulating the heterogeneity of single-cell metabolic phenotypes. This may enhance precision medicine approaches, drug development strategies or (eco)toxicological assessments. Besides the biomedical field, the proposed methodology is also of general interest as it provides a powerful framework for systematically characterizing O_2_ metabolism and its size-related fluctuations in other single-cell scenarios, ranging from the photosynthetic activity of marine microorganisms to the evaluation of the impact of chemicals or environmental stressors on single-cell respiration.

## Data availability

All the data supporting this manuscript, the source code of the multi-parameter identification algorithm and scripts for statistical analysis are available upon reasonable request to the corresponding authors.

## Author contributions

Conceptualization: AA, LB, AR, RS. Methodology: EB, YC, CM, MT, KK, LB, AA. Investigation: EB, YC. Visualization: EB, YC. Supervision: AA, LB, CM. Writing – original draft: EB. Writing – review & editing: All authors.

## Conflicts of interest

There are no conflicts to declare.

## Supplementary Material

LC-024-D4LC00204K-s001
