# Supplementary material for: Size-related variability of oxygen consumption rates in individual human hepatic cells
Source: Lab Chip. 2024 Jul 26;24(17):4128–37. doi: 10.1039/d4lc00204k (PMC11334764; doi:10.1039/d4lc00204k)
Supplement: LC-024-D4LC00204K-s001 [file LC-024-D4LC00204K-s001.pdf]

Supplementary Information (SI) for Lab on a Chip.
This journal is © The Royal Society of Chemistry 2024

Electronic Supplementary Information for

Size-related variability of oxygen consumption rates in individual human hepatic cells

Ermes Botte *et al.*

*Corresponding author. Email: arti.ahluwalia@unipi.it

This PDF file includes:

Supplementary Text
Figures S1 to S6
Table S1
ESI References

Supplementary Text

ESI1 – Characterization of the homogeneity of optode deposition

The bottom of the microwells was probed using profilometry [1] before and after application of the optode film to quantify its homogeneity and evaluate the reproducibility of the deposition process. A standard contact profilometer (Dektak 150 surface profiler, Bruker Nano Analytics, Billerica, Massachusetts, USA) based on the deflection of a diamond-tipped cantilever was used to determine the spatial distribution of the effective thickness as point-by-point height differences. The assessment was repeated for 21 microwells in six independently decorated devices, resulting in an overall effective thickness of 5 ± 3 µm (expressed as mean ± standard deviation for all assessed microwells). Most of variability in thickness was observed around the edges of the slide where optode accumulation predominates. For this reason, calibration and single-cell respiration measurements were conducted as far as possible from the edge of the array and each decorated microwell device was calibrated independently.

ESI2 – Non-linear optode calibration

Due to the availability of multiple quenching sites on dye molecules, the optical response of optode materials is better captured by the two-site model (Eq. (S1)) [2], rather than the standard Stern-Volmer equation [3].

$$\frac{R}{R_0} = \frac{f_1}{1 + k_{SV,1}O_2} + \frac{f_2}{1 + k_{SV,2}O_2} \qquad \text{(S1)}$$

where $k_{SV,i}$ indicates the SV constant of the i-th quenching site and f_i is the fraction of R_0 associated with the light emission of that site, so that $\sum_i f_i = 1$. As widely reported, we assumed that quenching occurring at the secondary site is negligible for PtTFPP [4], *i.e.*, $k_{SV,2} \rightarrow 0$. Considering $k_{SV,1} = k_{SV}$ and $f_2 = 1 - f_1 = 1 - f$, this ultimately leads to the simplified formulation in the main text (Eq. (2)). An example of the weighted fitting performed on five-point datasets to determine calibration parameters according to the simplified two-site model is reported in Figure S3A, showing the consistency of the above discussed assumptions.

It is worth noting that the non-linearity of the most of optode-based O_2 sensing – with a well-known higher sensitivity at low O_2 concentrations (*i.e.*, a super-linear behaviour) – makes the current sensor chemistry particularly suitable for biomedical applications.

ESI3 – Characterization of optode bleaching

O_2-sensitive optode materials like PtTFPP are reported to photo-bleach or produce singlet O_2 when exposed to excitation light for long times [5,6]. To compensate for potential artefacts arising from this phenomenon, optode responses were characterized over time, at multiple constant levels of air saturation (% air sat). Briefly, experimental measurements were performed by setting up the same five-point protocol used for calibration (see subsection *Calibration* in the main text). Optode responses were measured for 20 microwells over time, employing the same experimental parameters (Table 1, main text). The results of this optode bleaching experiment are summarized in Figure S3B. Sensor bleaching causes a very small measurement drift (expressed as percentage of the initially detected luminescence intensity ratio) limited to 1%/h across O_2 levels, increasing up to 2%/h only for 100% air saturation.

ESI4 – Characterization of optical crosstalk

Another aspect that could potentially interfere with the accuracy of O_2 concentration measurements is the optical crosstalk between luminescent optode materials deposited within adjacent microwells in the array. This may be caused by the radially directional spreading of light emitted by both the red O_2 sensitive dye (PtTFPP) and the green

reference dye (MY) as well as its scattering due to the surface roughness of microwells. To assess this contribution to the optical signal detected within microwells, we acquired images from 20 individual microwells exposed to excitation light (see Table 1 in the main text). Image acquisition was performed with a 40× objective under fully anoxic conditions, in order to measure the spatial characteristics of PtTFPP emission at maximum intensity, while all remaining parameters were identical to values provided in Table 1 in the main text.

The images were processed in Matlab (release R2021b). Given the isotropic nature of light scattering, polar symmetry was assumed, and the spatial decay of luminescence intensity (*I*) was modelled as a function of the radial coordinate (*r*) according to the following relationship:

$$I(r) = I(0)e^{-r/\delta} \qquad \text{(S2)}$$

where $I(0)$ is the light intensity measured at the edge of the microwell (corresponding to the arbitrarily chosen origin of the radial axis, see Figure S4), and δ (µm) denotes the characteristic radial distance, *i.e.*, the distance from the microwell edge at which light intensity is damped by a factor equal to $1/e$. Eq. (S2) was used to fit experimental profiles (Figure S4) and thus determine δ for both the red and green dye. More in detail, in both cases, δ was computed as the weighted average of those obtained by fitting the optical signal from each microwell.

$$\delta = \sum_{i=1}^{20} w_i \delta_i \qquad \text{(S3)}$$

In Eq. (S3), δ_i is the characteristic radial distance estimated for the *i*-th microwell, and w_i represents the associated weight, defined depending on the goodness of fit as follows:

$$w_i = \begin{cases} \sqrt{R^2{}_i \Big/ \sum_{i=1}^{20} R^2{}_i}, & R^2{}_i \geq 0.75 \\ 0, & R^2{}_i < 0.75 \end{cases} \qquad \text{(S4)}$$

where $R^2{}_i$ is the determination coefficient of the exponential fitting for the *i*-th microwell.

According to this rationale, we estimated characteristic distances δ_R = 51.7 µm and δ_G = 46.4 µm for PtTFPP and MY, respectively. Hence, if *d* denotes the inter-well distance (or, equivalently, the microwell diameter), considering design specifications reported in Table S1 and applying Eq. (S2) in the worst case (*i.e.*, PtTFPP under 0% O_2), at the edge of a neighboring well (*i.e.*, at *r* = *d*) the damping of luminescence intensity is given by $\frac{I(d)}{I(0)} \cong \frac{1}{e} \cong \frac{1}{3}$ for 50 µm diameter microwell arrays (*i.e.*, having *d* = 50 µm) and $\frac{I(d)}{I(0)} \cong \frac{1}{e^2} \cong \frac{1}{10}$ for 100 µm diameter ones (*i.e.*, having *d* = 100 µm). Based on these rough calculations, only adjacent wells with a diameter of 100 µm can be reliably assumed as optically decoupled (*i.e.*, $I(d) \ll I(0)$). Hence, in this study, devices with a microwell diameter of 100 µm were employed to measure the O_2 consumption of isolated cells.

ESI5 – <u>Optimization of oil-medium phase separation</u>

Finally, the optimal ratio between culture medium and heavy mineral oil used during experiments was evaluated. To this end, Nile Red (a lipophilic fluorescent dye with excitation wavelength 552 nm and emission wavelength 636 nm) was added to heavy mineral oil to obtain a 0.001% w/v solution, and images of the device closed with the coverslip were acquired to assess the homogeneity of the oil layer, while varying the quantities of both components. The best trade-off between culture medium and heavy mineral oil (Figure S5A) was obtained when using 60 µL of fresh DMEM dispensed onto the array and 60 µL of heavy mineral oil coating the coverslip. The plasma treatment - performed *a priori* – facilitated the filling of microwells with the aqueous phase (*i.e.*, DMEM), while heavy mineral oil tended to coat the inter-well spaces and overlay the culture medium (Figure S5A). The balance between the two is crucial. If the amount of heavy mineral oil is too low, part of the array was left uncovered (Figure S5C), reducing

the sealing effect. On the other hand, when too much oil was applied, it resulted in the displacement of DMEM (and cells, if any are present) out of the microwells (Figure S5B).

ESI6 – Dry mass measurements of human hepatic single cells

Single-cell dry masses of HepG2 cells were estimated using quantitative phase imaging (QPI). QPI leverages on physical principles of phase contrast microscopy, which exploits local phase shifts (or, equivalently, time delays) on reflected light waves. The shifts are induced by a specimen in the light path, and can hence be used for reconstructing its geometry, without application of any optical labels [7]. In fact, biological samples such as single cells or tissues do not damp the amplitude of electromagnetic fields in the visible range but are able to differentially shift their phase according to point-by-point variability in optical density, producing a phase map which can be compared to a proper reference for the image formation. In this study, a QPI camera system (SID4 sC8, PHASICS SA, Saint-Aubin, France) was integrated onto the same automated microscope used for characterizing single-cell O_2 consumption. To measure single-cell dry masses, 250 µL of a HepG2 cell suspension (at an arbitrary density) were dispensed onto a glass slide, closed with a coverslip and sealed with Scotch tape to avoid medium losses. Images were acquired for two different samples, either i) immediately after seeding or ii) after allowing cells to adhere to the glass during an overnight incubation. To obtain a reference for background subtraction, an area with no cells was first imaged, and subsequently individual cells were manually selected to assess their volume (and, thus, dry mass) via a proprietary software (sid4Bio, PHASICS SA) associated with the camera. All images were acquired using a dedicated 60× objective (Plan Apo – 60×/0.95, Nikon).

Collected datasets of HepG2 cell dry masses were processed using Matlab (release 2021b). Firstly, the Anderson-Darling (AD) test was performed on dry mass distributions of both suspended and adhered HepG2 cells (Figure S6A), demonstrating that the two distributions can be described by the same unspecified functional form (α = 0.01), corroborating the robustness of the approach with respect to isovolumetric changes of shape. This is coherent with cell incompressibility. In fact, mammalian cells are known to spread while adhering to the substrate, passing from an almost round to a flat shape [8]. The probability distribution of the obtained overall dataset - consisting of 272 measurements - was evaluated by means of a Lilliefors normality test performed on log-transformed cellular dry masses (α = 0.01), which demonstrated that it yields a lognormal form (Figure S6B).

The measured probability distribution of cellular dry masses is in agreement with those obtained for unicellular organisms [9]. Although statistically uncorrelated, this outcome also agrees with the 3D lognormality observed for the joint size-metabolism distribution that we obtained with these cells in independent experiments (see subsection *Joint measurements of single-cell size and metabolism* in the main text).

ESI7 – Estimation of the proximity index and uptake coefficient for hepatic cells in microwells

To assess whether O_2 metabolism of single HepG2 cells follows the dependency on cell packing that we observed in 2D and 3D aggregates [10], we computed the *PI* of microwells for different values of N_{cell}. The PI is estimated using a method based on Sholl analysis [11,12] to give a quantitative indicator of the closeness of neighbouring cell nuclei. As reported in the main text (section *Modelling single-cell consumption*), from a modelling point of view, each microwell can be treated as a 0D system, *i.e.*, whose dynamics only depends on time since it is spatially homogeneous. Should the microwell contain more than one cell, this implies that HepG2 cells are assumed to be homogeneously distributed within the domain. We thus introduced a lumped parameter, *L*, which describes the characteristic spatial dimension of the microwell as the cubic root of its volume (*i.e.*, $L = \sqrt[3]{\pi r_{well}{}^2 h_{well}}$). Based on the same lower limit and step considered in ref. [10], *L* can be used to determine both the number of distances (N_d) and range of distances (d) for Sholl analysis as N_d = 6 and $d \in$ [5; 55] µm, respectively. *PI* was then defined as in our previous study [10], so that we obtained the stepwise function in Eq. (S5):

$$PI = \begin{cases} \varepsilon^2, & N_{cell} = 1 \\ \frac{1}{N_{cell}} \sum_{i=2}^{N_{cell}} \left(\frac{1}{N_d} \sum_{j=1}^{N_d} \frac{n_{ij}}{d_j} \right), & N_{cell} > 1 \end{cases} \quad \text{(S5)}$$

where Matlab's machine epsilon ε = 10^{-16} expresses $PI \rightarrow 0$ for a single cell, and n_{ij} denotes the number of neighbouring cells detected within a distance d_j from the *i*-th cell. Estimating *PI* according to Eq. (S5) allowed for verifying whether corresponding uptake coefficients ($\varphi = sOCR\ PI/k_M$) extrapolated from the trend in [10] match those obtained using values of *sOCR* and k_M measured in this study.

Under the above-discussed hypotheses, the value of *PI* = 0.005 ± 0.003 µm^{-1}, which gives φ = 1.42 ± 0.90 fm^2 s^{-1}. Both values (expressed as median ± range for both parameters) are in line with the exponential trend observed in [10], displaying a steep decay of φ when *PI* < 0.01 µm^{-1}.

ESI References

1 T. Provder and B. Kunz, *Prog. Org. Coatings*, 1996, **27**, 219–226.
2 E. R. Carraway, J. N. Demas, B. A. DeGraff and J. R. Bacon, *Anal. Chem.*, 1991, **63**, 337–342.
3 M. Quaranta, S. M. Borisov and I. Klimant, *Bioanal. Rev.*, 2012, **4**, 115.
4 S. S. Lehrer, *Biochemistry*, 1971, **10**, 3254–3263.
5 J. Franck and R. Livingston, *J. Chem. Phys.*, 2004, **9**, 184.
6 J. Widengren and R. Rigler, *Bioimaging*, 1996, **4**, 149–157.
7 Y. K. Park, C. Depeursinge and G. Popescu, *Nat. Photonics 2018 1210*, 2018, **12**, 578–589.
8 A. A. Khalili and M. R. Ahmad, *Int. J. Mol. Sci.*, 2015, **16**, 18149.
9 A. Giometto, F. Altermatt, F. Carrara, A. Maritan and A. Rinaldo, *Proc. Natl. Acad. Sci. U. S. A.*, 2013, **110**, 4646–50.
10 E. Botte, P. Mancini, C. Magliaro and A. Ahluwalia, *APL Bioeng.*
11 D. A. Sholl, *J. Anat.*, 1953, **87**, 387.
12 C. Magliaro, A. L. Callara, N. Vanello and A. Ahluwalia, *Front. Neuroinform.*, 2017, **11**, 36.

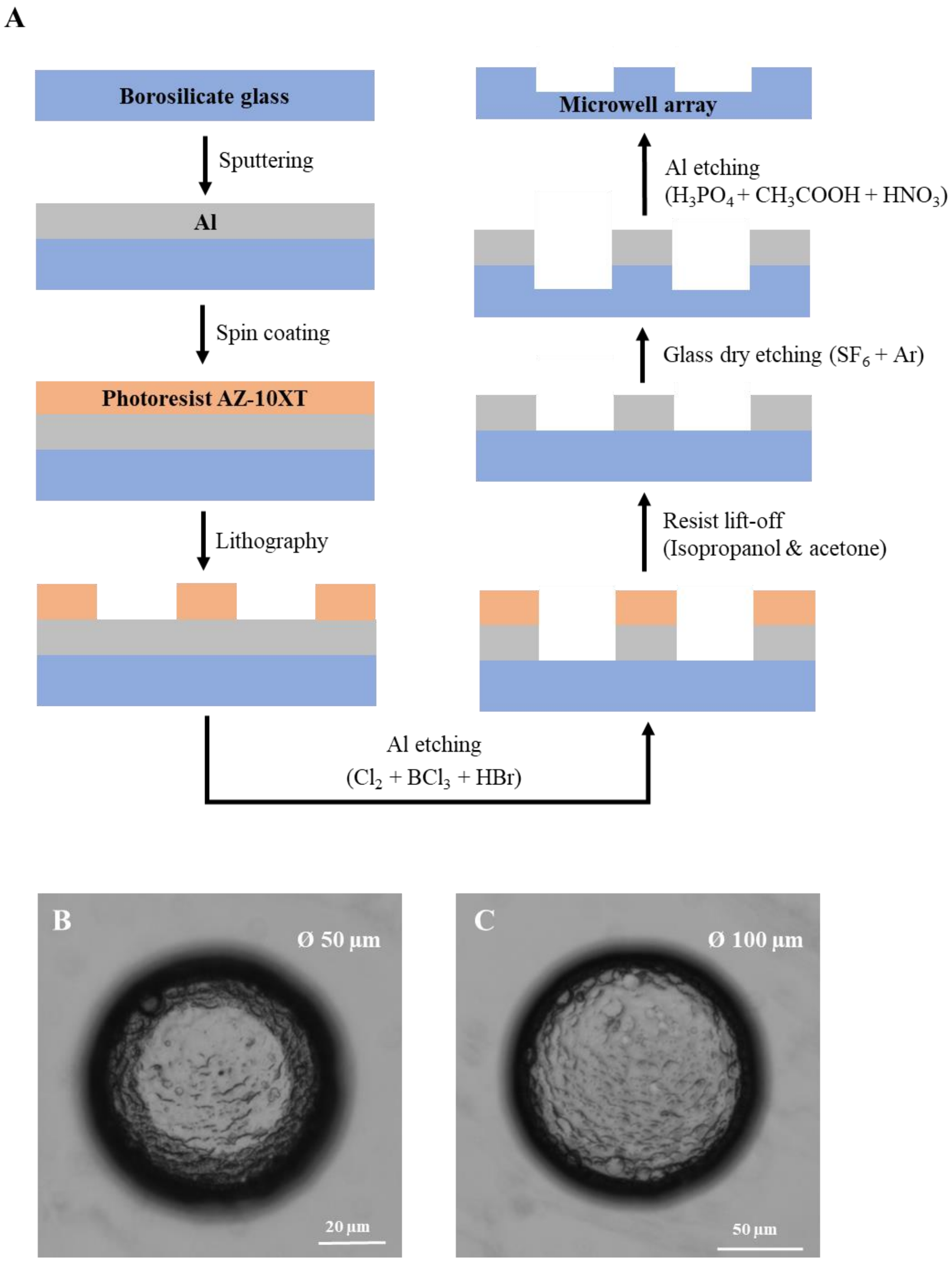

Figure S1. Microfabrication procedure of a microwell array for performing high-throughput single-cell isolation and readouts. (**A**) Based on the desired geometry, a custom aluminum mask for glass dry etching is realized using standard lithographic process and chemically removed after etching is completed. As a proof of the outcome, brightfield microscopy images of (**B**) a 50 µm-sized (40× objective) and (**C**) a 100 µm-sized (20× objective) microwell immediately after fabrication are shown.

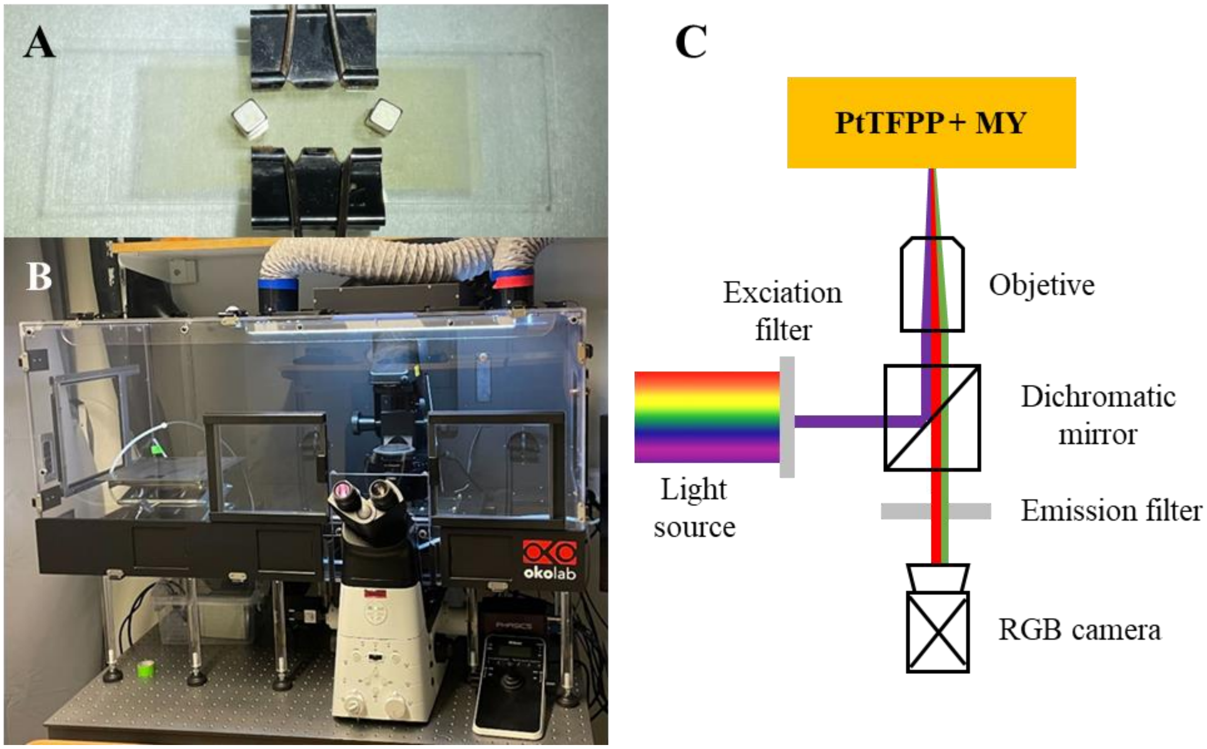

Figure S2. The experimental set up for performing single cell O_2 consumption measurements in the microwell array. (A) The custom-built glass microwell array**. (B)** The automated microscope surrounded by the transparent incubator. **(C)** Schematic of the optical working principle of the system.

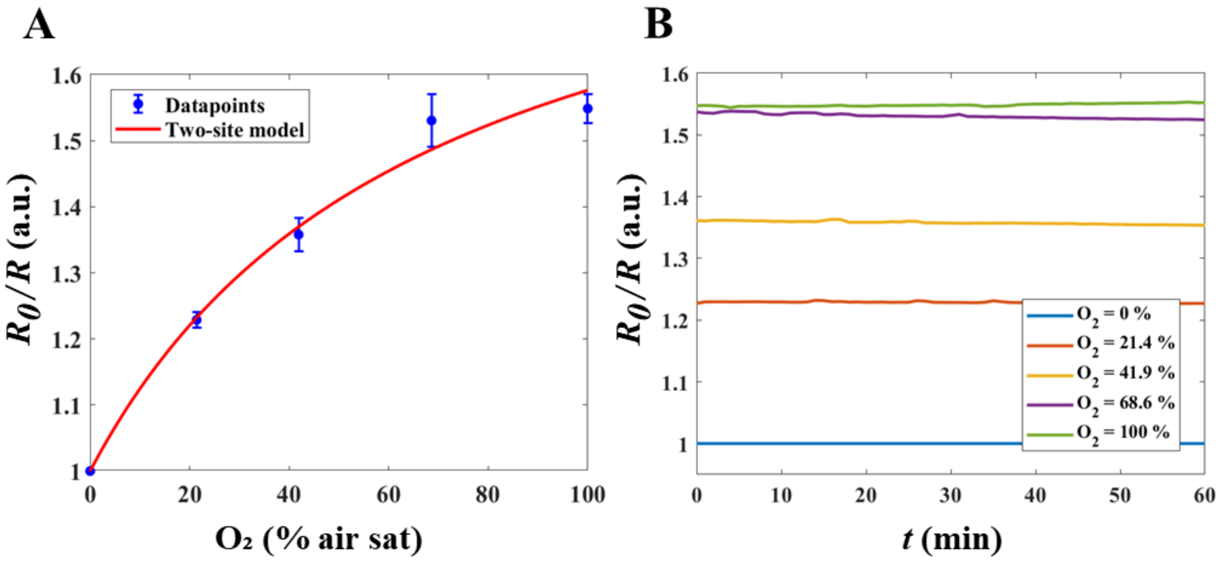

Figure S3. Non-linear calibration and characterization of optode bleaching. (A) An example of the weighted fitting performed on the five-point calibration dataset of a microwell array according to the simplified two-site model to estimate calibration parameters ($\boldsymbol{k_{SV}}$ = 0.029 1/% air sat and $\boldsymbol{f}$ = 0.491 with $\boldsymbol{R^2}$ = 0.993 in this specific case). Datapoints are expressed as median ± range. **(B)** Average luminescence intensity ratio measured over time on 20 microwells, parametrized with respect to the environmental air saturation as set through the gas mixer.

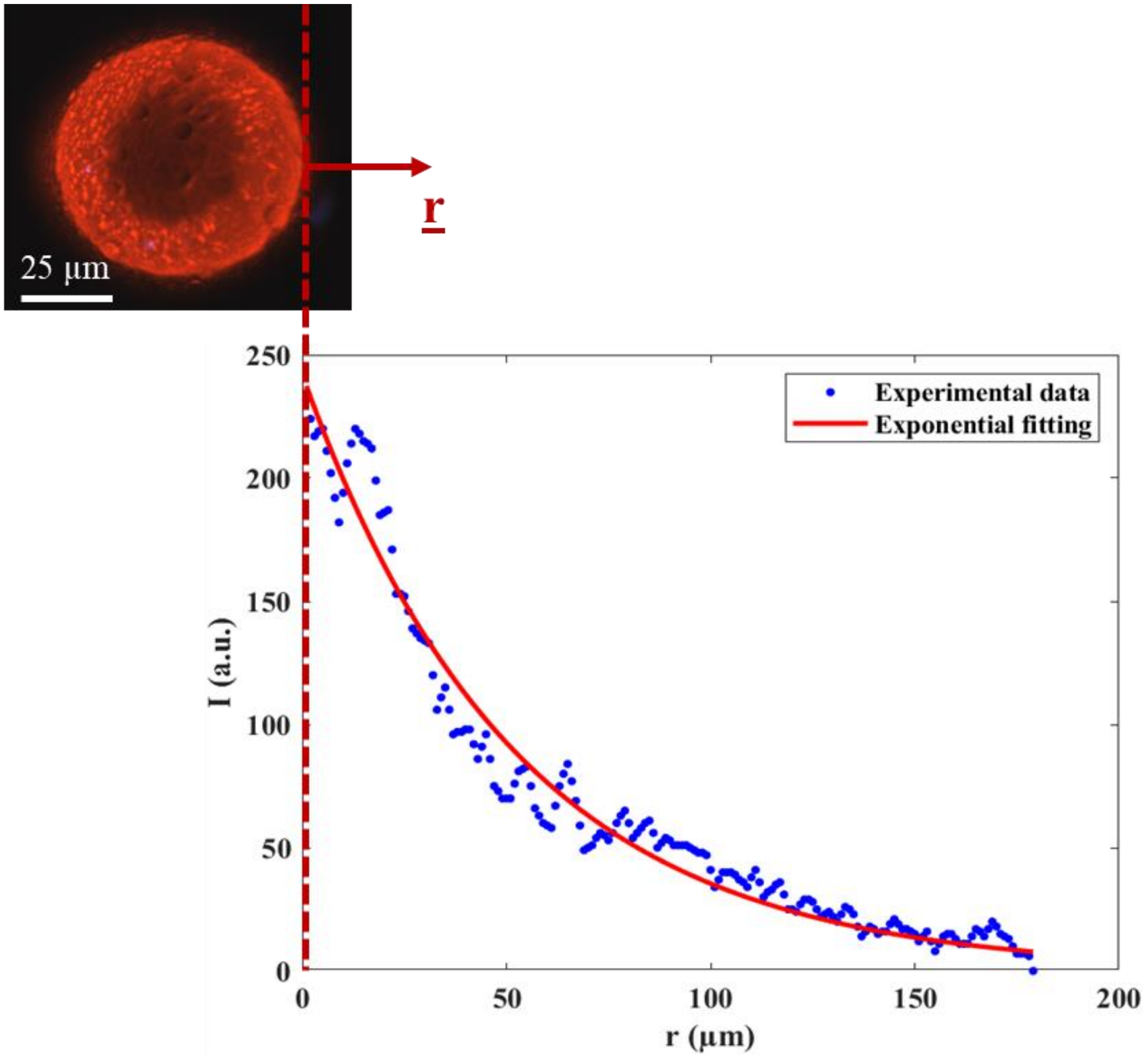

Figure S4. An example of the exponential fitting performed on luminescence intensity data for characterizing inter-well optical crosstalk. Light intensity is expressed as gray levels of the 8-bit grayscale image corresponding to the red or green channel of the RGB image of a single microwell (top left). The radial axis considered is shown in the small image above the intensity axis.

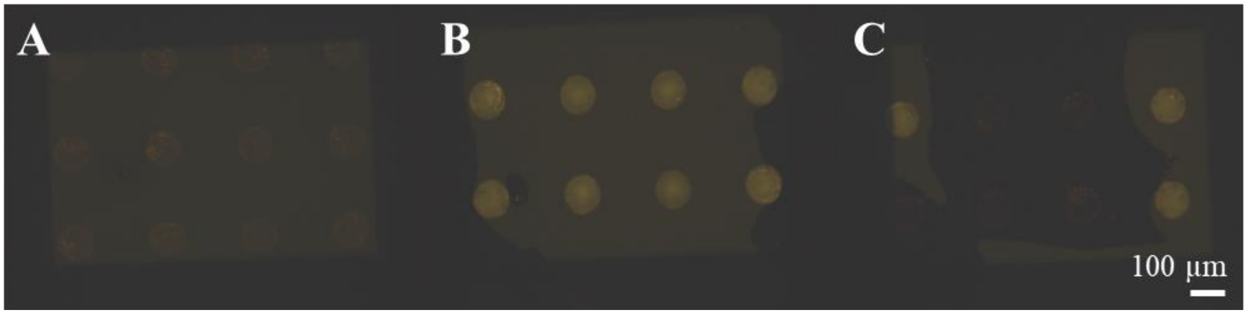

Figure S5. Heavy mineral oil with Nile Red for the optimization of medium-oil phase separation. (A) Ideal case of microwells filled with DMEM and overlayed with a homogeneous layer of heavy mineral oil (60 µL each). **(B)** Non-ideal case of microwells filled with heavy mineral oil as medium is displaced (30 µL of DMEM, 60 µL of oil). **(C)** Undesired case of a heterogeneous layer of heavy mineral oil, with some microwells left unsealed (60 µL of DMEM, 30 µL of oil). All images were acquired with a 10× objective and refer to an array with 100 µm-sized microwells.

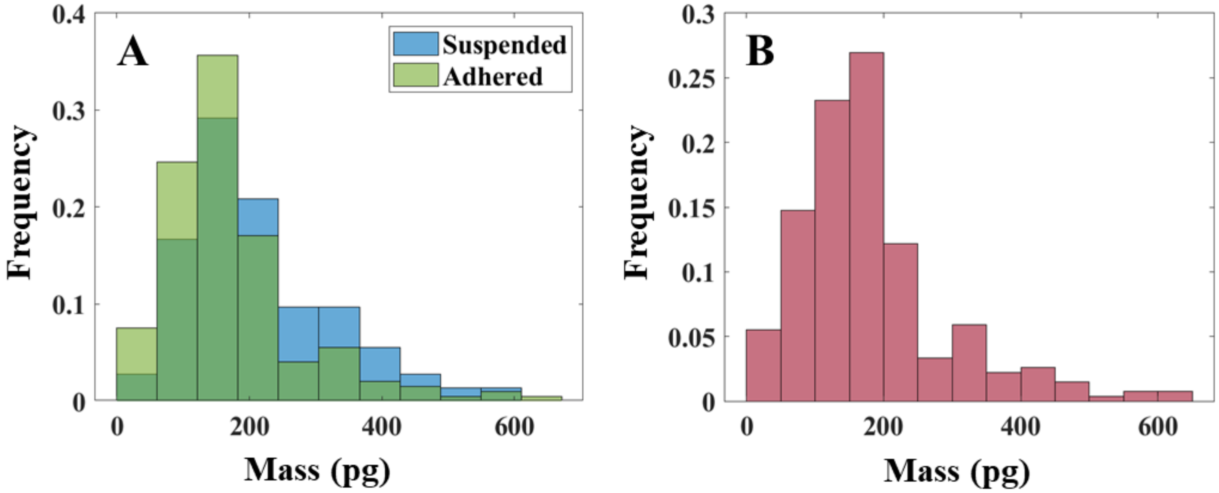

Figure S6. Dry mass distributions of HepG2 cells measured via a QPI approach. (A) Mass distributions from images acquired before (suspended, 73 cells) and after (adhered, 199 cells) incubation overnight. The AD test confirmed that their shape cannot be statistically distinguished (α = 0.01, p = 0.0226). **(B)** Overall experimental mass distribution (272 cells), yielding a lognormal form according to the Lilliefors test (α = 0.01, p = 0.0351). All histograms are in terms of relative frequencies.

Table S1. Technical features of the dry etching process and geometric specifications of the microwell array designed for measuring O_2 concentration over time in isolated HepG2 cells.

Parameter	Numerical value
Radial resolution	1 µm
Well diameter	50 or 100 µm
Well depth	20 µm
Well volume	39.3 pL
Well-well distance*	Same of diameter
Bottom roughness**	350 ± 150 nm

**Refers to the distance between the edges of adjacent microwells along the radial direction. **Refers to the arithmetic average roughness value [1] of the bottom surface for an etching depth of 20 µm, expressed as median ± range.*
